# 110 Years of change in urban tree stocks and associated carbon storage

**DOI:** 10.1002/ece3.1017

**Published:** 2014-03-20

**Authors:** Daniel F Díaz-Porras, Kevin J Gaston, Karl L Evans

**Affiliations:** 1 Department of Animal and Plant Sciences, University of SheffieldSheffield, S10 2TN, U.K; 2 Escuela de Ciencias, Universidad Autónoma ‘Benito Juárez’ de OaxacaOaxaca, 68120, Mexico; 3 Environment and Sustainability Institute, University of ExeterPenryn, Cornwall, TR10 9FE, U.K

**Keywords:** Carbon storage, ecosystem services, large trees, old trees, repeat photography, street trees, urban greening, urban tree planting, urbanization

## Abstract

Understanding the long-term dynamics of urban vegetation is essential in determining trends in the provision of key resources for biodiversity and ecosystem services and improving their management. Such studies are, however, extremely scarce due to the lack of suitable historical data. We use repeat historical photographs from the 1900s, 1950s, and 2010 to assess general trends in the quantity and size distributions of the tree stock in urban Sheffield and resultant aboveground carbon storage. Total tree numbers declined by a third from the 1900s to the 1950s, but increased by approximately 50% from the 1900s–2010, and by 100% from the 1950s–2010. Aboveground carbon storage in urban tree stocks had doubled by 2010 from the levels present in the 1900s and 1950s. The initial decrease occurred at a time when national and regional tree stocks were static and are likely to be driven by rebuilding following bombing of the urban area during the Second World War and by urban expansion. In 2010, trees greater than 10 m in height comprised just 8% of those present. The increases in total tree numbers are thus largely driven by smaller trees and are likely to be associated with urban tree planting programmes. Changes in tree stocks were not constant across the urban area but varied with the current intensity of urbanization. Increases from 1900 to 2010 in total tree stocks, and smaller sized trees, tended to be greatest in the most intensely urbanized areas. In contrast, the increases in the largest trees were more marked in areas with the most green space. These findings emphasize the importance of preserving larger fragments of urban green space to protect the oldest and largest trees that contribute disproportionately to carbon storage and other ecosystem services. Maintaining positive trends in urban tree stocks and associated ecosystem service provision will require continued investment in urban tree planting programmes in combination with additional measures, such as revisions to tree preservation orders, to increase the retention of such trees as they mature.

## Introduction

It is important to document temporal changes in urban green space and its associated vegetation, because of the rapidly expanding and dynamic nature of urban areas, and the key role of this vegetation in supporting urban biodiversity and providing ecosystem services (Seto et al. 2012; Gaston et al. 2013). Trees, particularly large ones, are keystone structures in many ecosystems, including urban areas (Lindenmayer et al. 2012; Stagoll et al. 2012). In towns and cities, the abundance and nature of trees plays a major role in determining the structure and composition of faunal assemblages (Evans et al. 2009; Stagoll et al. 2012). Trees and shrubs also play a key role in providing ecosystem services in urban areas, primarily because they comprise a considerable proportion of the vegetation's biomass (Davies et al. 2011; Roy et al. 2012). These benefits include a range of cultural services and improvements to human health and well-being (Ulrich 1986; Kuo and Sullivan 2001; Maas et al. 2006; Fuller et al. 2007). Urban vegetation also provides several regulating services including reducing air pollution (Donovan et al. 2005), the urban heat island effect (Lindberg and Grimmond 2011; Hall et al. 2012), noise pollution (Islam et al. 2012), and flood risk (Stovin et al. 2008). Finally, urban trees make a significant contribution to carbon sequestration (Nowak and Crane 2002).

Urban trees have historically faced a number of threats, and will continue to do so. Heat and drought stress seem likely to be amplified in urban areas due to the urban heat island effect, reduced water infiltration into soils due to the dominance of impervious surfaces, and soil compaction (Sieghardt et al. 2005). The urban heat island effect can also contribute to increased susceptibility of urban tree to pests (Meineke et al. 2013). Urban trees may also suffer more from pests and exotic diseases than their rural counterparts due to increased exposure to horticultural trade, for example, Asian long-horned beetle *Anoplophora glabripennis* became established in North America in urban areas and has only recently invaded rural ones (Dodds and Orwig 2011). Whilst air pollution can reduce growth rates of urban trees, there are some examples of increased growth rates in response to higher CO_2_ concentrations in urban areas (Evans 2010). Finally, urban trees are also more likely to be prevented from reaching their full growth potential due to the association between height and the probability of damaging urban infrastructure or blocking light.

Empirical data assessing changes in the nature and composition of urban green space are typically limited to use of remote-sensing data (e.g., Pauleit et al. 2005; Dallimer et al. 2011; Gillespie et al. 2012). Due to the timing of the development of appropriate technologies, such studies are inevitably restricted to a few recent decades; this is a small time period relative to the age of many urban areas, and assessments over longer-time periods are essential to provide a complete understanding of the impacts of urbanization. In addition, remote-sensing technologies have not always had sufficient capacity to distinguish individual components of green space, such as trees and shrubs, or to record their size. Given the strong relationship between ecosystem service provision and vegetation biomass and thus tree size (see above), this further limits assessment of the dynamics of urban vegetation. Collections of historical photographs provide a valuable source of detailed data on past environmental conditions that can be used to track long-term environmental change, which overcomes these limitations (Pennisi 2013). This approach is time-consuming as it requires finding a large number of dated historical images that include the key items of interest, and then refinding the original location from which these images were taken. Repeat photography has great value, however, and has been used to assess rates of glacial retreat, and changes in plant growth rates, vegetation composition, and forest cover (Chen et al. 2011; Myers-Smith et al. 2011; Van Bogaert et al. 2011). Such studies have rarely focused on urban areas, although Nowak (1993) used historical photographs in combination with other historical documents to assess vegetation change in Oakland, California. Monge-Nájera and Pérez-Gómez (2010) also used repeat photography to assess change in tree cover in San Jose, Costa Rica, but could only find nine suitable historical images.

Here, we employ repeat photography to assess long-term changes in the number and size of trees over a 110-year period using Sheffield, the fifth largest urban area (*c*. 555,500 people; Office for National Statistics 2010) in the UK, as a case study. We then use these data to assess temporal change in the contribution of the urban tree stock to aboveground carbon storage. We also test whether the temporal dynamics in the stock of urban trees is uniform across the urbanized region, or varies with the intensity of urban development. This is important because urban areas are not homogenous (Davies et al. 2008), and the magnitude and intensity of change can vary with urban form.

## Methods

### Obtaining and repeating historical photographs

We used a paired design and compared photographs taken in the 1900s and 1950s with those taken in 2010, although the two sets of historical images were not taken in the same location. Our objective was to calculate broad trends in the numbers of trees of different size categories to generate an index of change in urban tree stocks. Urban Sheffield was defined as those 1 × 1 km squares with at least 25% hard surface. Historical photographs were obtained from Sheffield's Local Studies Library online database (http://www.picturesheffield.com), which contains approximately 35,000 images, primarily from the 1900s. All images taken between 1900 and 1909 (referred to as the 1900s) or between 1950 and 1959 (referred to as the 1950s) were selected. The 1900s is the earliest decade for which sufficient images were available, and the 1950s represents a period of intense urban development following the Second World War.

**Figure 1:**
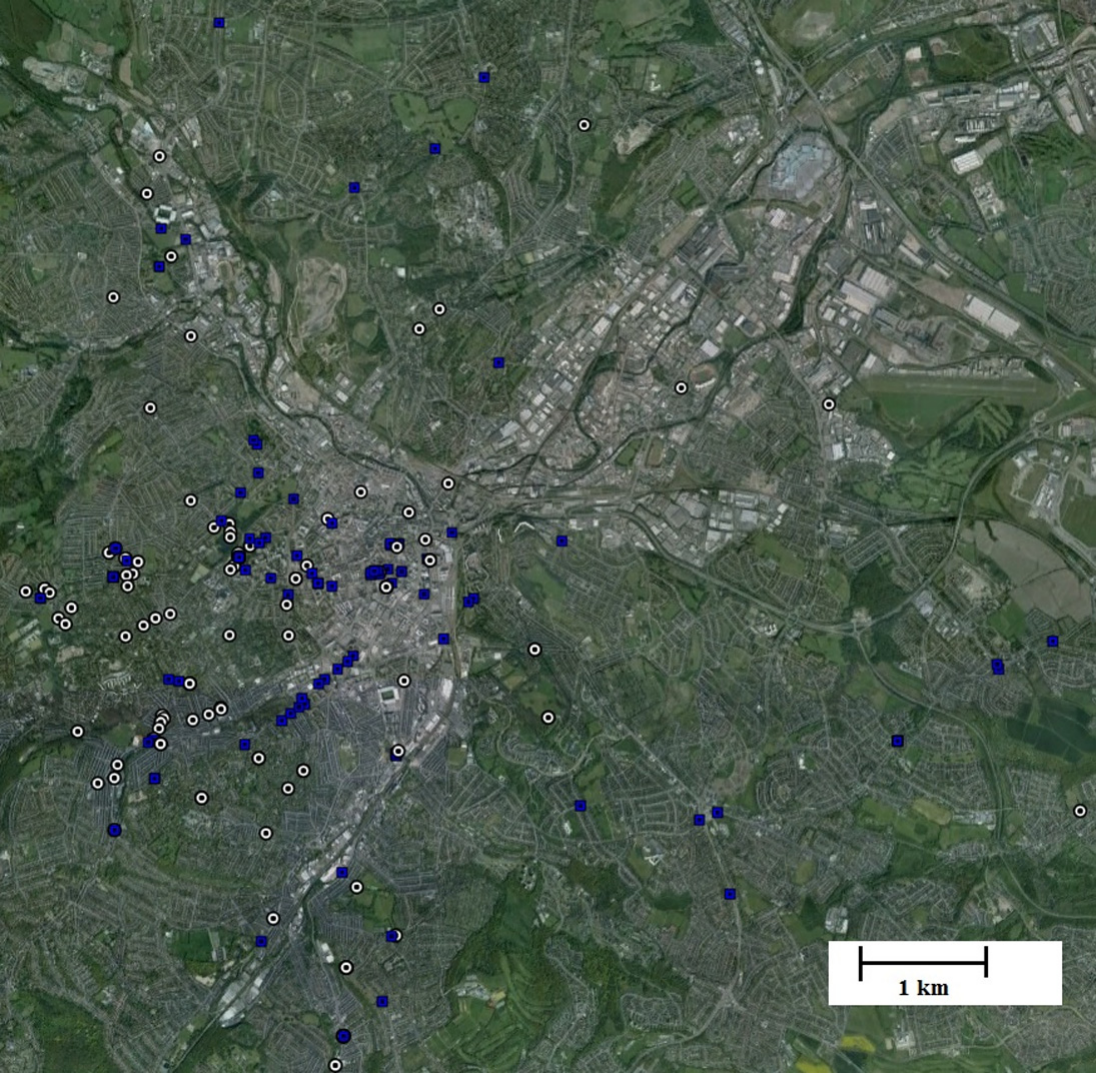
The location of the historical photographs from the 1900s (white circles) and the 1950s (blue squares) in urban Sheffield. Base imagery is from Google Earth and comprises a composite of images taken in 2008 and 2011.

We consider the set of historical photographs to represent an unbiased haphazard sampling design that is sufficient for estimating general trends in the urban tree stock for three reasons. First, the original photographic locations seem highly unlikely to have been selected on the basis of their tree cover. This is because the primary reason for taking the photographs was to record people or buildings – often both (e.g., photos of people taken outside their homes or work places). The massive variation in tree cover recorded in the historical images is one indication that positive or negative biases toward including trees in the historical images are unlikely to be large. Second, the locations of the historical images cover much of the focal urban region of Sheffield, albeit with an inevitable concentration in older urban areas that were urbanized in the 1900s and 1950s, and represent the full range of variation in urban form as assessed by the amount of green space currently present in the area (Fig. 1; and see Results). Finally, it seems unlikely that the location of the historical images would be biased according to future trends in tree cover as these were unknown at the time the images were taken.

Aerial images and those that mainly comprised the inside of buildings or obscured views were excluded. The potential to obtain a current image at precisely the same location as the historical image was assessed using the street view tool of Google Earth using three criteria: (1) the ability to use features in the historical image to pinpoint its exact location, (2) that the historic landscape captured in the original image was not currently obscured, and (3) that the site was accessible. When the potential could not be assessed using the street view tool (e.g., inside large parks), a site visit was conducted. Following these processes, 121 and 109 images were selected for the 1900s and 1950s, respectively. Additional searches were made for images from unrepresented boroughs taken during the contiguous decades, that is, within the 1890s and 1910s for the 1900s, and within the 1940s and 1960s for the 1950s. This resulted in a selection of 17 and 24 additional photographs, respectively, for 1890–1919 and 1940–1969. The former is hereafter referred to as the 1900s (88% of images are from 1900–1909) and the latter as the 1950s (82% of images are from 1950–1959).

Fieldwork was carried out from June to early September 2010. Repeat photographs were taken using a 4.6× optical zoom digital camera (12.2 megapixels) and matched the position and direction of historical photographs as closely as possible. Each photographic location was geo-referenced using a GPS. About 61 of the 271 historical photographs could not be repeated due to a failure to find the precise location of the original image or because the precise historical view could not be reconstructed. This left 106 pairs comparing the 1900s with 2010, and 104 pairs comparing the 1950s with 2010.

### Quantifying changes in the tree stock

All shrubs and trees present in the entire photograph were identified using the following height categories: (1) <2 m, (2) 2–5 m, (3) 5–10 m, and (4) >10 m. This was achieved by comparing the heights of individual trees and shrubs, by eye, with standardized reference heights of other features typically present in the urban landscape that were measured in the field; in addition, people were assumed to be <2 m tall. Whilst use of these reference heights does not provide a precise measure of the height of focal trees or shrubs, it provides an unbiased mechanism that can be applied to both historical and current time periods with which each shrub/tree can be accurately placed within a height category.

Aboveground dry-weight tree biomass was calculated using the allometric equation from Davies et al. (2011): biomass (kg) = 0.566*(height in meters)^2.315^, and summing across the total number of trees in each height category. When our height categories were bounded, we used their midpoint as an estimate of tree height, for the unbounded category of trees >10 m, and we repeated calculations using a range of tree height estimates (12, 15, and 18 m) that cover the full range of plausible midpoints based on observed size distributions of urban trees in the U.K. (Davies et al. 2011). The allometric equation that we used was developed for broad-leaved trees in urban Leicester, located 90 km south of Sheffield and of similar urban form. This equation takes into account the relative abundance of different tree species, and uses species, genus, or family-specific allometric relationships. This approach was adopted as historical photographs were rarely of sufficient quality to allow trees to be identified to species or genus. This will reduce the precision of our estimates of tree biomass as there may be some shifts in composition of the tree assemblage across time periods, but it does not prevent us from generating sufficiently accurate estimates to calculate overall trends in tree biomass and resultant carbon storage. This is because the form of allometric equations is fairly similar across different broad-leaved tree species, and broad-leaved trees comprised the vast majority of shrubs and trees in the historical and repeated images. This concurs with the regional and national pattern (Britt and Johnston 2008), and additional data collected as part of biodiversity surveys in Sheffield, that found that broad-leaved trees comprised 92.8% of trees. The five commonest tree species were sycamore *Acer pseudoplatanus*, ash *Fraxinus excelsior*, pedunculate oak *Quercus robur*, silver birch *Betula pendula,* and cherry *Prunus spp*. These data were obtained in 2010 from 140 sampling points selected using a random stratified design with regard to the amount of green space as described by Bonnington et al. (in press). We thus consider that our calculations provide a reasonably robust estimate of relative temporal change in tree biomass. Aboveground tree biomass (kg) was transformed to a carbon storage figure using the broadleaf conversion factor of 0.48 (Milne and Brown 1997).

### Calculating the percentage of green space

We wished to assess how trends in urban tree cover varied across different urban forms, which is most frequently measured by the amount of green space, or its inverse the amount of hard surface present in a given area. To achieve this, the amount of green space (i.e., vegetated surface, the majority of which is grass) currently present in the 250 × 250-m grid cell surrounding each photographic location was calculated using an OS Master 1:10000 scale Georeferenced TIFF raster map for the 2005–2009 period obtained from the Digimap Ordnance Survey Collection (via http://edina.ac.uk).

### Statistical analyses

All analyses were conducted using SPSS 16 (SPSS Inc., Chicago, IL) or SAS vs 9.3 (SAS Institute Inc., Cary, NC). We have two sets of paired photographs (1900s and 2010; 1950s and 2010), and the primary focus was to exploit this paired experimental design. We thus used a matched paired *t*-test to compare the urban tree stock (total number of trees, and numbers in each height category) that was present in the 1900s with that present in 2010, and to compare the tree stock in the 1950s with that present in 2010 (data on differences in the number of trees did not differ from a normal distribution; Kolmogorov–Smirnov test, *P *>* *0.05 in all cases). Photographic locations were different in the 1900s and 1950s and thus do not involve a paired design, and differences in the number of trees in these time periods did not follow a normal distribution. Changes in the urban tree stock between the 1900s and 1950s were thus analyzed using Mann–Whitney *U*-tests.

The percentage change in the number of shrubs/trees was calculated (for the total number of trees and for each height category except for trees > 10 m, see below) by adding one to the number of trees present to enable percentages to be calculated at sites with no trees. Percentages were then square-root transformed to meet statistical assumptions of normality; transformations were conducted on absolute values, and following transformation values that were originally negative were multiplied by minus one to preserve their original sign. We then used general linear models to model the transformed percentage change in the number of shrubs/trees as a function of the percentage of green space currently present in the surrounding 250 × 250-m grid cell. We did so using general linear models that include both linear and square terms as predictors, but removed the square term from the final model unless it was statistically significant (*P *<* *0.05). When the square term was included in the final model, we conducted a break point regression to assess the nature of the relationship between the percentage increase in shrubs/trees and green space below and above the turning point of the quadratic model. Moran's I values were consistently very low (<0.01 for all response variables) indicating that the data contained negligible spatial autocorrelation.

## Results

### 1900s–2010

**Figure 2:**
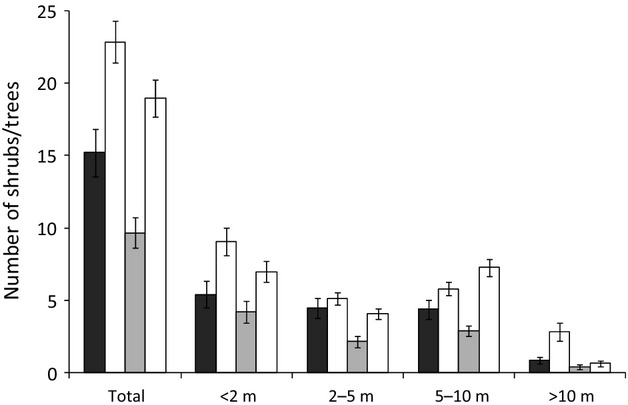
The number of shrubs and trees in urban Sheffield present in the 1900s (dark grey bars), 1950s (pale grey bars), and 2010 (white bars). Data are from 106 paired repeat photographs taken in the 1900s and 2010 (left-hand white bar in each category), and 104 paired repeat photographs taken in the 1950s and 2010 (right-hand white bar). Error bars represent standard errors.

The total number of shrubs/trees increased by 50.5% (*t *=* *6.20, df = 105, *P *<* *0.001; df = 105 in all cases; Fig. 2). Most size categories also exhibited significant increases: <2 m (67.6%, *t *=* *4.06, *P = *0.0001), 5–10 m (33.4%, *t *=* *2.01, *P = *0.05), >10 m (214.7%, *t *=* *3.36, *P = *0.0001), but the 13.7% increase in the number of shrubs/trees between 2–5 m was not significant (*P *=* *0.39; Fig. 2). Aboveground carbon storage in trees approximately doubled from the 1900s–2010, with the rate of increase being little influenced by the choice of midpoint for the unbounded height category (i.e., trees > 10 m; Table 1A).

**Table 1:** Change in aboveground carbon storage of the urban tree stock in Sheffield (U.K.) from (A) 1900 to 2010, and (b) 1950 to 2010. Biomass is calculated using the allometric equation for broad-leaved trees in urban Leicester (U.K.) from Davies et al. (2011) and converted to carbon storage following Milne and Brown (1997). Data are calculated using the summed number of trees present in historical and repeated photographs in four height categories (<2 m, 2–5 m, 5–10 m, > 10 m), and using the midpoint of each height category. Ratios of change are broadly consistent regardless of the midpoint used for the largest unbounded height category.

Height midpoint used for trees > 10 m	Aboveground tree carbon (kg) 1900	Aboveground tree carbon (kg) 2010	Carbon ratio (2010:1900)
(A)			
12 m	18142.8	35426.6	1.95
15 m	22599.2	48853.5	2.16
18 m	28399.6	66330.1	2.34
(B)			
12 m	13663.1	29804.4	2.18
15 m	16209.6	33682.1	2.08
18 m	19524.1	38729.2	1.98

### 1950s–2010

The total number of shrubs/trees increased by 95.8% (*t *=* *6.91, df = 103, *P *<* *0.001; df = 103 in all cases; Fig. 2). Most size categories also exhibited significant increases: <2 m (65.8%, *t *=* *3.05, *P = *0.003), trees between 2–5 m (88.8%, *t *=* *4.12, *P = *0.001), trees between 5–10 m (151.2%, *t *=* *7.24, *P = *0.001); the 52.3% increase in the number of trees > 10 m was not significant (*P *=* *0.30; Fig. 2). From the 1950s–2010, aboveground carbon storage in trees approximately doubled, with the choice of midpoint for the unbounded height category again having little influence on the estimated rate of change (Table 1B).

### 1900s–1950s

The total number of shrubs/trees declined by 37.5% (*U* = 4416.0, *P *=* *0.01). The numbers of shrubs/trees in each of the height categories also tended to decline during this period, but these differences were only significant for trees between 2–5 m in height (53.2%, *U* = 4066, *P *< 0.001), with other differences not being significant: <2 m (23.7%, *U* = 5079, *P *=* *0.295), 5–10 m (35.1%, *U* = 4942, *P *=* *0.175), and >10 m (53.7%, *U* = 5083, *P *=* *0.119).

### Relative abundance by height class in 2010

Pooling data from both sets of locations of historical images revealed that, across the 3598 trees captured, 36% were <2 m tall, 22% were 2–5 m tall, 34% were 5–10 m tall, and 8% were greater than 10 m in height.

### Relationships between changes in tree stocks and amount of green space

**Figure 3:**
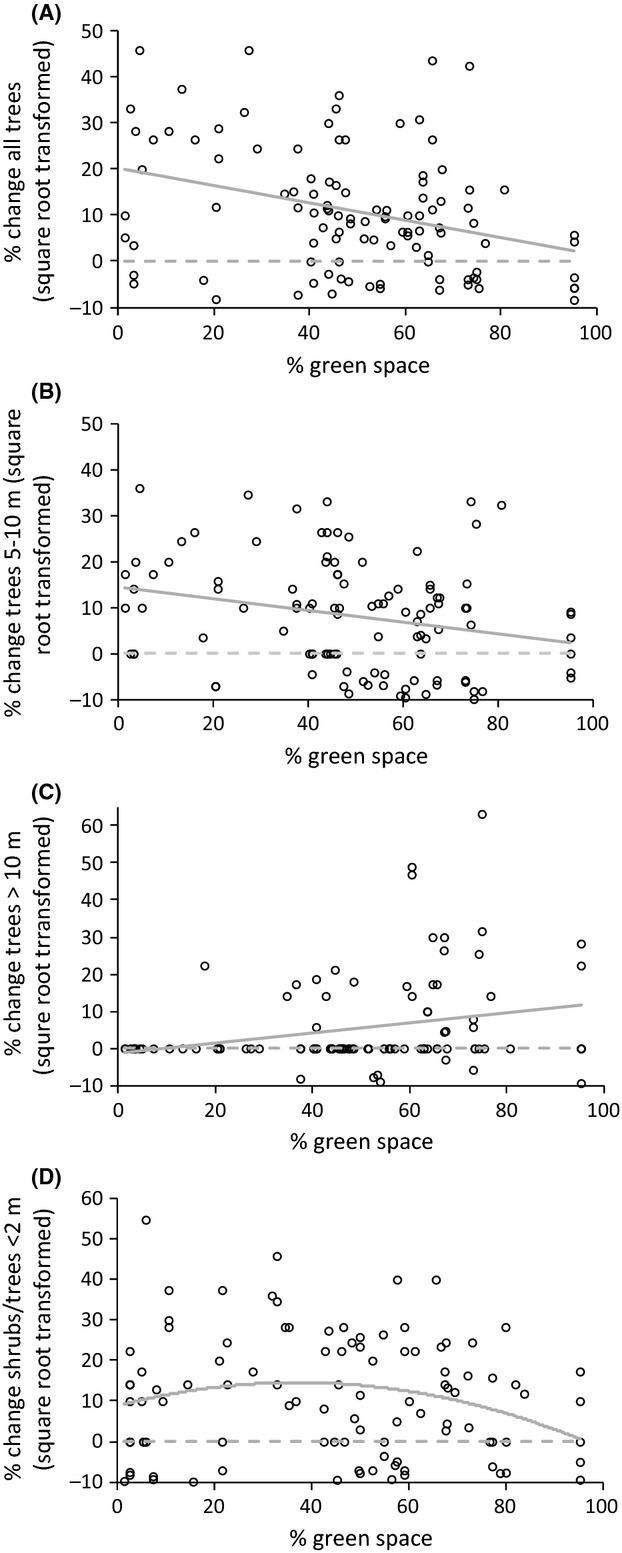
Relationships between the percentage increase in shrubs/trees and the amount of green space in the surrounding 250 × 250-m grid cell for (A) all shrubs/trees between the 1900s and 2010, (B) trees that are 5–10 m tall between the 1900s and 2010, (C) trees >10 m between the 1900s and 2010, and (D) trees <2 m between the 1950s and 2010.

Between the 1900s and 2010, the percentage increase in the total number of shrubs and trees and of trees between 5 m and 10 m tall was negatively associated with the amount of green space in the surrounding 250 × 250-m grid cells (Fig. 3A,B; Table 2A). The percentage increase in shrubs/trees that were <2 m and 2–5 m tall exhibited the same trend, but this was not statistically significant (Table 2). In contrast, the percentage increase in the number of trees that were taller than 10 m was greatest in areas that currently contained the most green space (Table 2A; Fig. 3C). Between the 1950s and 2010, the percentage increase in shrubs/trees that were <2 m tall exhibited a unimodal relationship with green space (no other relationships were statistically significant; Table 2B). Using a break point regression around the turning point of this unimodal relationship revealed that there was a significant positive association between the percentage increase in shrubs/trees that were <2 m tall until green space exceeded c. 40% of the surrounding 250 × 250-m grid cell (*r*^2^ = 15.5%; *F*_1,40_ = 7.34, *P *=* *0.01; parameter estimate 0.539 ± 0.200), after which the percentage increase in shrubs/trees was not associated with the amount of green space (*r*^2^ = 0.015%; *F*_1,60_ = 0.94, *P *=* *0.34; parameter estimate −0.110 ± 0.114).

**Table 2:** Relationships between percentage change in tree stocks in urban Sheffield from (A) the 1900s–2010, and (B) the 1950s–2010 in repeated historical photos and the amount of current green space in the surrounding 250 × 250-m grid cell. The percentage change in tree stocks was square-root transformed prior to analysis. All data refer to linear terms unless otherwise indicated.

Height class	Model *r*^2^, %	Parameter estimate (±SE)	*F* ratio; *P* value	Equation
(A)				
All trees	10.99	−0.189 ± 0.053	*F*_1,104_ = 12.84; P = 0.0005	*Y* = 20.302 −0.189*x*
<2 m	0.25	−0.029 ± 0.056	*F*_1,104_ = 0.26, *P *=* *0.609	n/a
2–5 m	2.54	−0.081 ± 0.049	*F*_1,104_ = 2.71, *P *=* *0.103	n/a
5–10 m	6.32	−0.128 ± 0.048	*F*_1,104_ = 7.02, *P *=* *0.009	*Y* = 14.580−0.128*x*
>10 m	6.76	0.134 ± 0.049	*F*_1,104_ = 7.54, *P *=* *0.007	*Y* = −1.345 + 0.134*x*
(B)				
All trees	2.71	−0.103 ± 0.061	*F*_1,102_ = 2.84; P = 0.095	n/a
<2 m	4.92	Linear term: 0.307 ± 0.175 Square term: −0.004 ± 0.002	Linear term *F*_1,101_ = 0.79, *P *=* *0.082; Square term *F*_1,101_ = 4.43, *P *=* *0.034;	*Y* = 8.831 + 0.307*x* - 0.004*x*^2^
2–5 m	0.67	−0.035 ± 0.043	*F*_1,102_ = 0.69, *P *=* *0.409	n/a
5–10 m	3.13	−0.083 ± 0.046	*F*_1,102_ = 3.29, *P *=* *0.073	n/a
>10 m	0.12	−0.009 ± 0.023	*F*_1,102_ = 0.13, *P *=* *0.718	n/a

## Discussion

We demonstrate that repeat photography can yield valuable data for long-term monitoring of urban tree stocks, and associated ecosystem services. Between the 1900s and 2010, shrubs/trees within urban Sheffield increased by over 50%. Equivalent studies conducted over comparable time periods are rare, and none have been conducted in regions with the long history of urbanization that characterizes our study, which further hinders direct comparisons. It is notable though that studies conducted in regions where forest cover is naturally limited, such as South-West North America, tend to find increased urban tree cover. In Oakland, California, for example, tree cover increased from approximately 5% during the city's initial development (1850s–1890s) to approximately 20% in 1991 (Nowak 1993). Similarly, tree densities more than doubled from the 1920s to the turn of the century at two urban sites near Los Angeles, California, although a small number of urban areas had decreased tree cover (Gillespie et al. 2012). In contrast, a 5% decrease in urban tree cover occurred from the 1890s–2010 in San José, Costa Rica (Monge-Nájera and Pérez-Gómez 2010): a region that naturally has a high level of forest cover.

The significant increase in the number of urban shrubs/trees in Sheffield since the 1900s is thus not unprecedented, but does represent one of the most marked rates of increase documented to date. One factor that may contribute to this is that in the early 1900s, past human activities had reduced tree cover across England to just 6%, and to less than 4% across Yorkshire, the county in which Sheffield is located (Forestry Commission 2001). The increase in total shrubs/trees was even more marked (c. 100%) from the 1950s–2010, due to a decrease in urban tree cover in the first half of the twentieth century which contrasts with a static trend in tree cover at the national level across this time period (Forestry Commission 2001). This decrease from 1900 to 1950 in urban tree abundance is likely to be a consequence of the marked urban intensification during this period, and bombing (and associated redevelopment) during the Second World War. The pattern that we find in Sheffield is similar to the initial trends in urban tree cover that arose in Baltimore, Maryland, with an initial decrease from 1914 to 1938, which was then followed by an increase till the 1970s (Zhou et al. 2011). There has subsequently been a decline in urban tree cover in Baltimore, resulting in no net change from 1914 to 2004. It should thus not be assumed that the increase in urban tree cover that we document in Sheffield will be maintained in the future, especially given the numerous and increasing threats to urban trees that seem likely to increase mortality rates (see Introduction).

We find clear evidence that small trees, that is, those less than two meters tall, are now commoner in urban Sheffield than they were in both the 1900s (68% increase) and 1950s (66% increase). Natural seedling abundance and establishment is lower in urban woodlands than rural ones, suggesting that natural regeneration is suppressed in urban areas (Oldfield et al. 2013). It thus seems likely that the increase in small trees since the 1900s and 1950s is at least partly driven by urban tree planting initiatives. Whilst explanatory power is somewhat limited, there is a tendency for smaller trees to exhibit larger increases in abundance in the areas with least green space, that is, the most intensively urbanized areas. This strengthens the conclusion that urban tree planting programmes have contributed to the increase in the number of small trees, as natural regeneration is likely to be particularly low in such sites.

The increase in the number of trees from the 1950s–2010 becomes larger as tree size increases from <2 m (66%), to 2–5 m (89%), and to 5–10 m (150%). There is insufficient data on the annual height increments of broad-leaved trees in urban environments to estimate robustly the age of these trees. Growth rates of *Prunus*,*Acer,* and *Quercus* species growing in rural areas of the UK (Willoughby 2009), at similar climatic conditions in rural Belgium (Ligot et al. 2013) and in urban North America (Dereli et al. 2013), suggest though that annual growth rate increments will vary from c. 20 cm per year for slower growing species such as *Quercus* to 40 cm per year for other faster growing species. These growth rates suggest that urban tree planting schemes that were most frequent in the UK in the 1970s and 1980s (Land Use Consultants 1993; Urban Green Spaces Task Force 2002; Britt and Johnston 2008) could also have contributed to the increased abundance of trees in the 2–5 m and 5–10 m height categories from the 1950s–2010.

The major increase (c. 200%) in the largest trees (>10 m) that occurred from 1900 to 2010 was much less pronounced from 1950 to 2010. This could imply that mortality/removal of larger trees have increased in recent decades, but it also could arise from some variation in the number of larger trees found in 2010 at the locations of the historical photos from the 1900s and 1950s. The occurrence of such stochastic variation is partly driven by the extreme rarity of trees greater than 10 m tall; they account for just 8% of urban trees in 2010. The typical height of mature broad-leaved trees in the UK is much greater than 10 m, for example, ash 20 m, sycamore 24 m, oak 30 m (Fitter and Peat 1994). These three species were the commonest species in Sheffield in 2010 (see Introduction). Our data thus strongly suggest that urban regions are particularly deprived of large old trees, but we still find increases in recent time periods. Moreover, we find a tendency for the largest trees to exhibit greater rates of increase in the areas with most green space, that is, the least urbanized sites. This is presumably because the negative impacts of large urban trees, such as root damage to buildings and street surfaces and the blocking of light, are less likely to occur in the least urbanized sites. It is particularly important to maintain these large trees because of the crucial role they play in providing wildlife resources (Stagoll et al. 2012), cultural ecosystem services (Jim 2004), and their disproportionate contribution to provisioning and regulating services due to their increased biomass (Akbari et al. 2001; Davies et al. 2011).

Space-for-time substitutions (Pickett 1989) are often used in urban ecology to assess the consequences of increasing urbanization intensity over time. The associations we find between rates of increase in tree numbers and urbanization intensity suggest that spatial urbanization gradients may not always provide a reliable measure of change along temporal urbanization gradients. This has important implications for the use of space-for-time swaps in urban systems.

Our data suggest that investment in urban tree planting programmes has contributed to the increase in the number of urban trees over our focal 110-year time period. Maintaining investment in such programmes is thus advisable. This has been achieved in recent years through the Big Tree Plant Campaign which aims to plant an additional one million, mainly urban, trees in England between 2010 and 2015 (http://www.defra.gov.uk/bigtreep lant), but future commitments are uncertain. Moreover, we find some evidence that the smallest trees have increased in abundance the most in areas with little green space, that is, those areas that we also find have the lowest rates of growth in larger trees, which is probably a consequence of increased mortality, for example, tree removal to limit damage to urban infrastructure. Urban tree planting programmes may thus make a larger contribution to future long-term increases in the abundance of old and large trees by giving extra consideration to the potential of planting sites to maintain such trees. Larger trees also contribute disproportionately to ecosystem services, and a more comprehensive and holistic assessment of their benefits may reduce removal rates in situations when tree-associated damage is small relative to the benefits provided by the focal tree. Tree preservation orders in North America have been successful in protecting urban tree stocks when supported by sufficient investment in management and enforcement (Hill et al. 2010; Landry and Pu 2010). In the UK, tree preservation orders can only be applied to trees with high amenity value. This is not precisely defined, but is determined by the suitability of the trees for the focal site, their visibility, and impact, which is a function of factors such as their size, rarity, and screening potential (Department for Communities and Local Government 2006, 2012). Consequently, tree preservation orders are unlikely to be granted for trees in areas with little green space and thus a greater risk of damaging infrastructure or blocking light, or to smaller trees even when surrounded by lots of green space. Enabling preservation orders to be applied to such trees by considering their future rather than just their current amenity value seems likely to reduce tree mortality rates, and further increase the beneficial legacy of urban tree planting programmes by increasing the proportion of such trees that reach full maturity.
